# Effectiveness of the Fun for Wellness Web-Based Behavioral Intervention to Promote Physical Activity in Adults With Obesity (or Overweight): Randomized Controlled Trial

**DOI:** 10.2196/15919

**Published:** 2020-02-27

**Authors:** Nicholas D Myers, Adam McMahon, Isaac Prilleltensky, Seungmin Lee, Samantha Dietz, Ora Prilleltensky, Karin A Pfeiffer, André G Bateman, Ahnalee M Brincks

**Affiliations:** 1 Michigan State University East Lansing, MI United States; 2 University of Miami Coral Gables, FL United States

**Keywords:** eHealth, mHealth, self-efficacy theory, physical activity self-efficacy level, self-regulatory efficacy, mediation

## Abstract

**Background:**

Insufficient physical activity in the adult population is a global pandemic. Fun for Wellness (FFW) is a self-efficacy theory- and Web-based behavioral intervention developed to promote growth in well-being and physical activity by providing capability-enhancing opportunities to participants.

**Objective:**

This study aimed to evaluate the effectiveness of FFW to increase physical activity in adults with obesity in the United States in a relatively uncontrolled setting.

**Methods:**

This was a large-scale, prospective, double-blind, parallel-group randomized controlled trial. Participants were recruited through an online panel recruitment company. Adults with overweight were also eligible to participate, consistent with many physical activity–promoting interventions for adults with obesity. Also consistent with much of the relevant literature the intended population as simply adults with obesity. Eligible participants were randomly assigned to the intervention (ie, FFW) or the usual care (ie, UC) group via software code that was written to accomplish equal allocations to the FFW and UC groups. Data collection was Web based, fully automated, and occurred at three time points: baseline, 30 days after baseline (T2), and 60 days after baseline (T3). Participants (N=461) who were assigned to the FFW group (n_FFW_=219) were provided with 30 days of 24-hour access to the Web-based intervention. A path model was fit to the data consistent with the FFW conceptual model for the promotion of physical activity.

**Results:**

There was evidence for a positive direct effect of FFW on transport-related physical activity self-efficacy (beta=.22, *P*=.02; *d*=0.23), domestic-related physical activity self-efficacy (beta=.22, *P*=.03; *d*=0.22), and self-efficacy to regulate physical activity (beta=.16, *P*=.01; *d*=0.25) at T2. Furthermore, there was evidence for a positive indirect effect of FFW on physical activity at T3 through self-efficacy to regulate physical activity at T2 (beta=.42, 95% CI 0.06 to 1.14). Finally, there was evidence for a null direct effect of FFW on physical activity (beta=1.04, *P*=.47; *d*=0.07) at T3.

**Conclusions:**

This study provides some initial evidence for both the effectiveness (eg, a positive indirect effect of FFW on physical activity through self-efficacy to regulate physical activity) and the ineffectiveness (eg, a null direct effect of FFW on physical activity) of the FFW Web-based behavioral intervention to increase physical activity in adults with obesity in the United States. More broadly, FFW is a scalable Web-based behavioral intervention that may effectively, although indirectly, promote physical activity in adults with obesity and therefore may be useful in responding to the global pandemic of insufficient physical activity in this at-risk population. Self-efficacy to regulate physical activity appears to be a mechanism by which FFW may indirectly promote physical activity in adults with obesity.

**Trial Registration:**

ClinicalTrials.gov NCT03194854; https://clinicaltrials.gov/ct2/show/NCT03194854.

## Introduction

### Background

The objective of this study was to evaluate the effectiveness of the Fun for Wellness (FFW) intervention to increase physical activity in adults with obesity in the United States in a relatively uncontrolled (ie, real world) setting. The study described in this paper was conceptualized as an effectiveness trial (ie, participants were recruited via a national health care panel recruitment company) that built upon a 2015 FFW efficacy trial completed in a relatively controlled setting (ie, participants were recruited at a major research university in the United States) [1-3]. This study is important from a general scientific perspective because the potential utility of interventions should be evaluated under both more controlled (eg, scientifically ideal: an efficacy trial) and less controlled (eg, real-world ideal: an effectiveness trial) conditions [4,5]. Before describing the FFW intervention, we begin with a summary of the 2015 FFW efficacy trial and then introduce key components in this study: target population (ie, adults with obesity), proposed outcome (ie, physical activity) and mediator (ie, self-efficacy), and the theoretical framework (ie, self-efficacy theory).

### 2015 Fun for Wellness Efficacy Trial

A randomized controlled trial completed in 2015 provided the initial test of the efficacy of the FFW intervention to promote well-being [1-3]. The FFW intervention was conceptualized as exerting both a positive direct effect and a positive indirect effect through self-efficacy on well-being. Data collection occurred within a relatively controlled environment (ie, adult employees at a major research university in the United States). Results provided some initial evidence for the efficacy of FFW to promote well-being self-efficacy [3]; interpersonal, community, psychological, and economic subjective well-being [1]; and interpersonal and physical well-being actions [2]. The effectiveness trial described in this paper sought to follow up on the initial evidence provided in the 2015 FFW efficacy trial.

### Adults With Obesity

Approximately 2 billion adults are overweight per the World Health Organization (WHO) [6]. Moreover, approximately one-third of adults who are overweight can more precisely be classified as adults with obesity and the size of this subgroup has tripled over the past few decades [6]. In the United States, more than 40% of women and 35% of men are obese [7]. From a public health perspective, this trend toward an increasing number of adults with obesity is problematic because obesity is a risk factor for major noncommunicable chronic diseases such as cardiovascular disease, type II diabetes, musculoskeletal disorders, and some cancers [8]. To reduce the prevalence of adults with obesity, the WHO recommends that individuals increase energy intake from high-quality food sources (eg, raw vegetables), limit energy intake from low-quality food sources (eg, highly processed foods high in fat), and engage in a recommended amount of physical activity for health [6]. Examples of a recommended amount of physical activity for health in adults include at least 150 min per week of moderate-intensity physical activity or at least 75 min per week of vigorous-intensity physical activity, or an equivalent combination of the two recommendations listed above [9,10]. However, there is evidence that a very small percentage (eg, <5%) of adults with obesity meet the public health guidelines for physical activity [11]. Fortunately, there is also evidence that cognitive behavioral interventions can successfully promote physical activity in adults with obesity [12,13] and in the more general adult population [9].

### Physical Activity

Physical activity has been defined as bodily movement produced by skeletal muscles that requires energy expenditure [14]. Insufficient physical activity in the adult population is a global pandemic [15,16]. Successfully addressing this pandemic will require ongoing and wide implementation of a variety of intervention strategies (eg, community-wide, informational, behavioral, social, policy, and built environment) at multiple levels of society (eg, individual, neighborhood, municipality, and country) across the globe [17,18]. At the individual level, there is evidence that behavioral interventions designed to promote physical activity by focusing on personal psychological attributes (eg, self-efficacy) can be effective [19-21]. Delivering a physical activity intervention online has been shown to be an effective mode of delivery [22,23] that also may allow for efficient scaling up of an intervention [18]. Thus, a readily scalable, Web-based behavioral intervention that effectively promotes physical activity in adults with obesity may be useful in responding to a global pandemic (ie, physical inactivity) in an at-risk population (ie, adults with obesity).

### Self-Efficacy Theory

The social cognitive theory [24] has provided the theoretical framework for many effective cognitive behavioral physical activity–promoting interventions for adults with obesity [12,13]. Self-efficacy theory [25] resides within social cognitive theory and views an individual as a proactive agent in the regulation of their emotions, cognitions, and behaviors. Self-efficacy beliefs play a primary role in the self-efficacy theory and are defined as domain-specific judgments held by an individual about their ability to successfully execute differing levels of performance given certain situational demands. Self-efficacy beliefs rely upon the cognitive processing of several potential sources of efficacy information: enactive mastery experiences, vicarious experiences, verbal persuasion, and physiological and emotional states. Furthermore, two proposed omnibus outcomes of self-efficacy beliefs are an individual’s thought patterns (eg, goal setting, worry, and attributions) and behaviors (eg, challenges undertaken, effort expended on challenges undertaken, and persistence in the face of difficulties that arise during challenges undertaken). A necessary condition for valid testing of self-efficacy theory is concordance between the domain-specific self-efficacy beliefs and the proposed outcome of interest. There is a rich literature on the potential importance of targeting self-efficacy as a potentially modifiable mediating variable in physical activity–promoting interventions [19-21].

The self-efficacy theory posits that a self-efficacy–level construct may play a central role in the initiation of a behavior (eg, engaging in a recommended amount of weekly physical activity), whereas self-efficacy to regulate a behavior construct may play a central role in the maintenance of a behavior (eg, engaging in a recommended amount of weekly physical activity over time) [25]. A self-efficacy–level construct can be defined as an individual’s beliefs in his or her ability to accomplish levels of a task (eg, engage in at least 150 min of moderate-intensity physical activity in the next week). Self-efficacy to regulate a behavior construct can be defined as an individual’s beliefs to overcome possible barriers to accomplishing a task that he or she already knows how to do (eg, engage in at least 150 min of moderate-intensity physical activity in the next week if you are under personal stress). The importance of both a self-efficacy level construct and self-efficacy to regulate a behavior construct has been demonstrated in exercise contexts [26,27]. However, there still exists a pressing need to systematically test self-efficacy theory–based interventions to promote physical activity in real-world settings [5,9,21].

### Fun for Wellness

**Figure 1:**
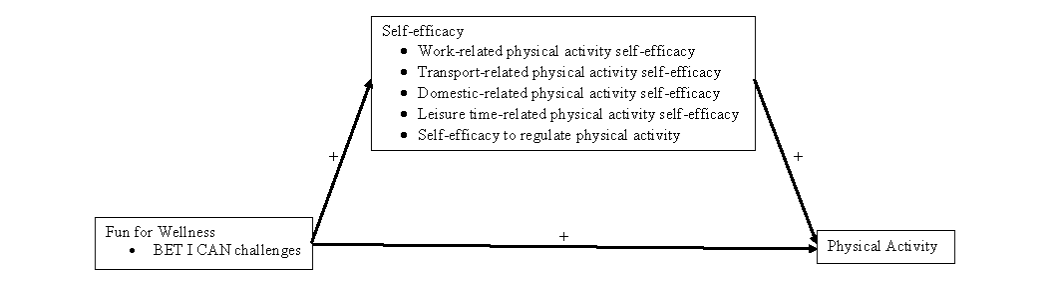
The Fun for Wellness conceptual model for the promotion of physical activity. BET I CAN: behaviors, emotions, thoughts, interactions, context, awareness, and next steps.

FFW is a self-efficacy theory–based, online (ie, Web-based and not an app) behavioral intervention developed to promote growth in well-being and physical activity by providing capability-enhancing opportunities to participants [28]. The full conceptual model for the FFW intervention is broader than this study and specifies that FFW exerts both a positive direct effect and a positive indirect effect through self-efficacy (ie, well-being self-efficacy, well-being action self-efficacy, physical activity self-efficacy, self-efficacy to regulate physical activity) on well-being (ie, subjective well-being, well-being actions, and physical activity). The narrower focus of this study was on the FFW conceptual model for the promotion of physical activity (see Figure 1). Consistent with the self-efficacy theory [24,25], the behaviors, emotions, thoughts, interactions, context, awareness, and next steps (BET I CAN) challenges provided in the FFW intervention (described in the next section) are specified as positive sources of self-efficacy information that exert a positive direct effect on self-efficacy beliefs, which are then specified to exert a positive direct effect on physical activity (ie, a behavior) [28]. Thus, self-efficacy is specified as a mediating variable in the FFW conceptual model for the promotion of physical activity.

#### Behaviors, Emotions, Thoughts, Interactions, Context, Awareness, and Next Steps Challenges

The self-efficacy theory provided the theoretical framework that guided the creation of capability-enhancing learning opportunities (ie, the BET I CAN challenges) with which FFW participants engage [1]. The capability-enhancing learning opportunities provided to participants exist in the form of 152 interactive and scenario-based challenges organized in the on-line environment by the BET I CAN acronym. The *B*ehavior-focused challenges are intended to increase a participant’s capabilities to set a goal and to create positive habits. The *E*motion-focused challenges are intended to increase a participant’s capabilities to cope with negative emotions and to cultivate positive emotions. The *T*hought-focused challenges are intended to increase a participant’s capabilities to challenge negative assumptions and to create a new narrative for their life. The *I*nteraction-focused challenges are intended to increase a participant’s capabilities to communicate and connect with others. The *C*ontext-focused challenges are intended to increase a participant’s capabilities to read cues and to change cues in the environment. The *A*wareness-focused challenges are intended to increase a participant’s capabilities to know themselves and to know the issue. The *N*ext steps–focused challenges are intended to increase a participant’s capabilities to make a plan and to stick with it. The scientific literature for each type of BET I CAN challenge has been reviewed elsewhere [28].

The capability-enhancing learning opportunity within each of the 152 BET I CAN challenges provides each FFW participant with exposure to one or more of Bandura’s potential sources of self-efficacy information [3]. More specifically, each BET I CAN challenge requires a participant to do one of the following activities: (1) play an interactive game, (2) watch vignettes performed by professional actors, (3) listen and read minilectures narrated by a coach, and (4) engage in self-reflection exercises and chat rooms. An opportunity for an enactive mastery experience is provided when a participant plays an interactive BET I CAN game. An opportunity for a vicarious experience is provided when a participant watches a BET I CAN vignette performed by professional actors. An opportunity to be verbally persuaded is provided when a participant listens to a BET I CAN minilecture narrated by a coach. An opportunity for assessing relevant physiological and emotional states is provided when a participant is asked to engage in a BET I CAN self-reflection exercise. The scientific literature supporting each of these proposed sources of self-efficacy information in physical activity contexts has been reviewed elsewhere [21,25,29].

#### Self-Efficacy Beliefs

Both a self-efficacy–level construct (ie, physical activity self-efficacy) and self-efficacy to regulate a behavior construct (ie, self-efficacy to regulate physical activity) are included in the FFW conceptual model for the promotion of physical activity [28]. Physical activity self-efficacy has been defined in the FFW context as the degree to which an individual perceives that they have the capability to engage in a recommended amount of weekly physical activity for health. Self-efficacy to regulate physical activity has been defined in the FFW context as the degree to which an individual perceives that they have the capability to overcome possible barriers to engagement in a recommended amount of weekly physical activity for health.

Both the physical activity self-efficacy construct and the self-efficacy to regulate physical activity construct were recently added to the FFW conceptual model based on two key results and limitations from the 2015 FFW efficacy trial [28]. First, although results from the 2015 FFW efficacy trial provided some initial evidence for the efficacy of FFW to promote physical well-being actions [2], measurement of physical well-being actions consisted of only 2 items (ie, how often do you engage in moderate physical activity such as brisk walking for about 30 min at least five times a week and eat mostly a plant-based diet of foods such as fruits, vegetables, nuts, and seeds). This study seeks to address this limitation by more thoroughly measuring physical activity across four general domains of life: leisure related, domestic related, work related, and transport related [30,31]. Second, although results from the 2015 FFW efficacy trial provided some initial evidence for the efficacy of FFW to promote self-efficacy [3], measurement of self-efficacy focused on well-being self-efficacy (ie, the degree to which an individual perceives that they have the capability to attain a positive status in key domains of their life) and, thus, was not very concordant with physical activity. This study seeks to address this limitation by more thoroughly measuring the self-efficacy beliefs for physical activity (ie, leisure-related, domestic-related, work-related, and transport-related physical activity self-efficacy and the self-efficacy to regulate physical activity).

### Hypotheses

In all, three construct-level a priori hypotheses were investigated in this study based on the conceptual model depicted in Figure 1. Hypothesis 1 was that the FFW intervention would exert a positive direct effect on self-efficacy. Hypothesis 2 was that self-efficacy would exert a positive direct effect on physical activity. Hypothesis 3 was that the FFW intervention would exert a positive direct effect on physical activity. An additional construct-level exploratory hypothesis (ie, hypothesis 4) was also investigated based on the conceptual model depicted in Figure 1: the FFW intervention would exert a positive indirect effect on physical activity through self-efficacy. Dimension-specific hypotheses were not made because of a lack of previous research on the effectiveness of the FFW intervention to promote physical activity.

## Methods

### The Well-Being and Physical Activity Study

The data described in this paper were collected within a more broadly focused trial, the Well-Being and Physical Activity Study (ClinicalTrials.gov, identifier: NCT03194854). Within this section, we provide an overview of the relevant methods used in the Well-Being and Physical Activity Study to provide a context for the specific focus of this paper [32]. The readers are referred to the relevant protocol paper [28] for a fuller description of the protocol for the Well-Being and Physical Activity Study. A populated Consolidated Standards of Reporting Trials-EHEALTH checklist is provided in Multimedia Appendix 1.

### Ethics Approval

All procedures in this study involving human participants were in accordance with the ethical standards of the institutional and national research committee and with the 1964 Helsinki declaration and its later amendments or comparable ethical standards. The institutional review board (IRB) at the University of Miami provided necessary permission to conduct this study on July 11, 2017, IRB number 20170541. The University of Miami and Michigan State University (STUDY00000979) established an Institutional Authorization Agreement on June 26, 2018, that provided permission for the University of Miami to serve as the designated IRB for this study.

### Study Design

The study design was a large-scale, prospective, double-blind (ie, investigators and outcome assessor were masked), parallel-group randomized controlled trial. Recruiting, screening, random assignment, and collection of data were conducted online from August 2018 through November 2018. Data collection was Web based, fully automated, and occurred at three time points: baseline (T1), 30 days after baseline (T2), and 60 days after baseline (T3). The timeline for this study was similar to timelines used in other physical activity interventions in adults with obesity [12,13].

### Recruitment and Eligibility

A sample size of approximately 900 participants was targeted for enrollment in the study. Participants were recruited through the general population panel of the SurveyHealth recruitment company. Partnering with a panel recruitment company is consistent with recruitment in preliminary research on FFW [33,34] and with a movement toward larger and smarter physical activity promotion interventions [18]. Eligibility criteria were (a) the ability to access the Web-based intervention, (b) living in the United States, (c) aged 18 to 64 years, (d) BMI of 25.00 kg/m^2^ or more, and (e) absence of simultaneous enrollment in another intervention program promoting either well-being or physical activity. The BMI criterion included both the overweight (ie, 25.00-29.99 kg/m^2^) category and the obese category (ie, ≥30.00 kg/m^2^) consistent with many physical activity–promoting interventions for adults with obesity [12,35].

### Informed Consent

Informed consent was obtained from each participant included in the study. More specifically, immediately after being determined to be eligible for this study, each eligible individual was directed to a Web-based, IRB-approved informed consent form. Each individual who clicked *Consent to Participate* was enrolled as a participant in the study. Each individual who clicked *Decline to Consent* was denied access to the study.

### Random Assignment

Random assignment of each eligible participant occurred after a unique and secure login credential was created, informed consent was obtained, a medical disclaimer was agreed to, and the T1 survey battery was completed. Eligible participants were randomly assigned to the intervention (ie, FFW) or the usual care (ie, UC) group via software code that was written to accomplish equal allocations to the FFW and UC groups. Participants assigned to the FFW group were given immediate access to the intervention. Participants assigned to the UC group were put on a waitlist for access to the intervention. Both the FFW group and the UC group were provided with modest financial incentives to provide data consistent with a general approach taken in many theory-based physical activity–promoting interventions [9]. The authors of this study are unaware of any previous research that would support casting unique doubt on the results of this study (as compared with other theory-based physical activity–promoting interventions that used modest financial incentives in a study) attributable to the particular financial incentives approach taken in this study.

#### Usual Care

Participants assigned to the UC group were asked to conduct their lives as usual. The login credential for each UC participant provided access to a secure website to complete the survey battery at T1, T2, and T3. UC participants had the opportunity to earn up to US $30 worth of Amazon electronic gift cards. Specifically, UC participants could earn US $5 for completing the T1 survey battery, US $10 for completing the T2 survey battery, and US $15 for completing the T3 survey battery. UC participants were given 1 month of 24-hour access to the FFW intervention after data collection for this study was closed.

#### Fun for Wellness

Participants assigned to the FFW group were asked to engage with the FFW intervention. The login credential for each FFW participant provided 30 days (ie, from T1 to T2) of 24-hour access to the 152 BET I CAN challenges and access to a secure website to complete the survey battery at T1, T2, and T3. FFW participants had the opportunity to earn a total of US $45 worth of Amazon electronic gift cards. Specifically, FFW participants could earn US $5 for completing the T1 survey battery, US $10 for completing both the T2 survey battery and at least 15 BET I CAN postintroductory challenges, an additional US $15 for completing at least 30 BET I CAN post-introductory challenges, and US $15 for completing the T3 survey battery.

Each of the first four BET I CAN challenges required the participant to do one of the aforementioned activities while focusing on introductory material (orientation to the website, examples of a recommended amount of physical activity for health, etc) to provide an important context for capability-enhancing opportunities [25]. Participants were required to complete these introductory challenges to gain access to the remaining 148 postintroductory BET I CAN challenges. Participants self-selected which postintroductory BET I CAN challenges to complete. Challenges completed by each participant were tracked by computer software to provide data (ie, participation points) for the FFW engagement scoring system [1]. Earning at least 21 participation points was the operational definition for being engaged with the FFW intervention [28].

### Survey Battery

Instruments designed to measure demographic information, self-efficacy, and physical activity were included in the survey battery. Proposed demographic and biological correlates of physical activity were collected via self-reporting at T1 and included participant age, gender, race/ethnicity, highest level of education completed, marital status, employment status, and household annual income [19]. This set of demographic and biological variables is collectively referred to as the demographic covariates from this point forward.

#### Physical Activity

Physical activity was measured at T1 through T3 with the long form of the international physical activity questionnaire (IPAQ [30,31]). The long form of the IPAQ is intended for individuals aged 15 to 69 years and purports to measure physical activity in four domains—work related, transport related, domestic related, and leisure time related—according to the frequency and duration of the physical activity performed in each domain during a week. The physical activity domains measured in the IPAQ are separated according to their intensity, which is defined as a distinction between walking, moderate physical activities, and vigorous physical activities. Moderate physical activity is defined as activities that take moderate physical effort and make you breathe somewhat harder than normal. Vigorous physical activity is defined as activities that take hard physical effort and make you breathe much harder than normal.

A total physical activity score—which is the sum of total walking time, total time in moderate physical activity, and total time in vigorous physical activity—was created based on the IPAQ data processing guidelines [36]. Total walking time is the sum of walking time in the work-related, transport-related, and leisure-related domains. Total time in moderate physical activities is the sum of moderate physical activity in the work-related, transport-related, domestic-related, and leisure-related domains. Total time in vigorous physical activities is the sum of vigorous physical activity in the work-related, domestic-related, and leisure-related domains. Outlying cases (ie, averaging 16 hours or more of physical activity per day) were excluded from analysis based on IPAQ data processing guidelines for excluding outliers [36].

#### Self-Efficacy

Overall, five domains of self-efficacy were measured at T1 through T3. Each of the four physical activity self-efficacy–level domains was measured with a slightly modified version of the well-established 8-item exercise self-efficacy (EXSE; [26]) scale. The EXSE scale assesses an individual’s beliefs in their ability to continue exercising on a 3-times-per-week basis at moderate intensities for more than 40 min per session in the future. The EXSE scale was tailored for the FFW context to assess the degree to which an individual perceives that they have the capability to engage in a recommended amount of weekly physical activity for health. Work-related physical activity self-efficacy was measured with a 12-item scale that was designed to be concordant with how work-related physical activity is measured in the IPAQ (ie, at both a vigorous and moderate intensity). Transport-related physical activity self-efficacy was measured with a 6-item scale that was designed to be concordant with how transport-related physical activity is measured in the IPAQ (ie, at a moderate intensity). Domestic-related physical activity self-efficacy was measured with a 6-item scale that was designed to be concordant with how domestic-related physical activity is measured in the IPAQ (ie, at a moderate intensity). Leisure-related physical activity self-efficacy was measured with a 12-item scale that was designed to be concordant with how leisure-related physical activity is measured in the IPAQ (ie, at both a vigorous and a moderate intensity). Vigorous-intensity items began with the stem “how confident are you in your current ability to engage in *work-* or *leisure*-related physical activity at a vigorous level of intensity” and then referenced six increasing periods (eg, for at least 10, 15, 30, 45, 60, or 75 min in the next week). Moderate-intensity items began with the stem “how confident are you in your current ability to engage in *work-* or *transport-* or *domestic-* or *leisure*-related physical activity at a moderate level of intensity” and then referenced six increasing time periods (eg, for at least 10, 30, 60, 90, 120, or 150 min in the next week). Responses to each item were organized within a 5-category rating scale structure, where 0=no, 1=low, 2=moderate, 3=high, and 4=complete confidence based on previous research on effective self-efficacy rating scale structures [37]. An average observed score for each of the four physical activity self-efficacy level domains was created based on relevant guidelines [26,38].

Self-efficacy to regulate physical activity was measured at T1 through T3 with a slightly modified version of the well-established 13-item barriers self-efficacy (BARSE) scale [27]. The BARSE scale assesses an individual’s perceived capabilities to exercise 3 times per week for 40 min over the next 2 months in the face of commonly identified barriers to participation. The BARSE scale was tailored for the FFW context to assess the extent to which an individual believes that he or she has the ability to overcome possible barriers to engagement in a recommended amount of weekly physical activity for health. Responses to each item were organized within a 5-category rating scale structure, where 0=no, 1=low, 2=moderate, 3=high, and 4=complete confidence. An average observed score for self-efficacy to regulate physical activity was created based on relevant guidelines [27,38].

### Data Analytic Approach

Statistical models were fit in Mplus 8.3 with maximum-likelihood (ML) estimation with robust SEs [39]. Type I error rate was set equal to 0.05. Missing data were addressed with full information ML estimation using the observed information matrix under the assumption of missing at random [40]. Reliability was assessed using Cronbach alpha [41,42]. Indexes of effect size considered for direct effects were Cohen *d* [43] and percentage of variance explained. Commonly used heuristics were used to assist in the interpretation of an absolute value of Cohen *d*: 0.20 (small), 0.50 (medium), and 0.80 (large). For each indirect effect, a bias-corrected bootstrapped estimate of the 95% confidence interval was obtained with the number of draws set equal to 2000 [44]. An index of effect size was not considered for indirect effects because an effect size index for complex mediation models has not yet been firmly established [45].

### Path Model

A single saturated (degrees of freedom=0) path model was fit consistent with the conceptual model depicted in Figure 1 under an intention-to-treat approach [46]. Each of the five domains of self-efficacy at T2 were regressed on FFW (ie, 0=UC, 1=FFW), physical activity at T1, and the demographic covariates. Physical activity at T3 was regressed on FFW, the five domains of self-efficacy at T2, physical activity at T1, and the demographic covariates. The expression *adjusted mean difference* is used from this point forward to acknowledge the statistical adjustment made by including covariates in the model.

There were four sets of focal parameters in the path model. The first set of focal parameters was the direct effect of FFW on each of the five domains of self-efficacy at T2 (ie, beta_1_). Each of these five parameters was interpreted as the adjusted mean difference on a particular domain of self-efficacy at T2 for the FFW group as compared with the UC group. The second set of focal parameters was the direct effect of the five domains of self-efficacy at T2 on physical activity at T3 (ie, beta_2_). Each of these five parameters was interpreted as the path coefficient from a particular domain of self-efficacy at T2 to physical activity at T3. The third set of focal parameters was a single parameter: the direct effect of FFW on physical activity at T3 (ie, beta_3_). This parameter was interpreted as the adjusted mean difference on physical activity at T3 for the FFW group as compared with the UC group. The fourth set of focal parameters was the indirect effect of FFW on physical activity at T3 through each of the five domains of self-efficacy at T2 (ie, beta_4_, where beta_4_=beta_1_*beta_2_'). Each of these five parameters was interpreted as the product of path coefficients from FFW to physical activity at T3 through a particular domain of self-efficacy at T2. Each set of focal parameters tested the numerically corresponding hypothesis (eg, beta_1_ tested hypothesis 1).

#### Necessary Sample Size

Necessary sample size was determined for a minimum fixed level of power (ie, 0.80) for rejecting the null hypothesis that each of the five focal parameters regarding a direct effect of FFW (ie, beta_2_ and beta_3_) was equal to 0.00 using Monte Carlo methods as implemented in Mplus 8.3 [47]. The population parameter value for each of the five relevant focal parameters was set equal to a value that corresponded to a small-to-moderate effect size (ie, *d*=.35) consistent with relevant results from previous research [2,3]. Type I error was set equal to 0.05. The number of replications was set to 10,000, and the necessary sample size was equal to 285.

## Results

### Participant Characteristics

**Figure 2:**
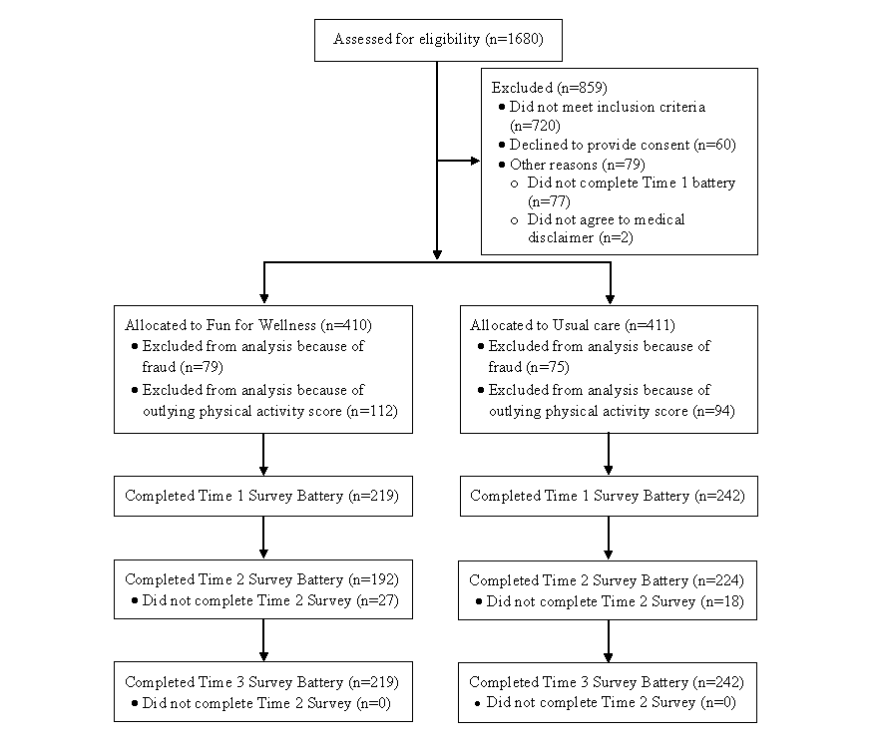
Participant flow from screening to randomization to retention over the three measurement occasions for the physical activity–related data.

Figure 2 depicts participant flow from eligibility screening to randomization to retention over the three measurement occasions. A total of 821 consenting participants were randomly assigned to FFW (n*=*410) or UC (n*=*411). Forensic analysis by a computer scientist performed before data analysis identified 154 cases as fraudulent, and these cases were excluded from analysis. The researchers initiated the forensic analysis after consulting with the designated IRB, legal counsel, and the office of research compliance and quality assurance about the computer scientist’s report of suspicious activity on the website (eg, participants logging in very close temporal proximity and sending identical emails to the computer scientist in broken English). The forensic analysis revealed that all of these 154 accounts were made by 1 user or group through 2 virtual private server (VPS) services. The analysis was reported as a reportable new information (RNI#00003760) incident to the designated IRB in December 2018. Unlike the 154 fraudulent cases (ie, 154/821, 18.8%), no groupings of the 667 nonfraudulent cases (667/821, 81.2%) appeared to have been made by 1 user or group through VPS services.

An additional 206 cases were outlying cases on the physical activity score and were excluded from the analysis, leaving 461 analyzed cases (ie, participants), FFW (n*=*219) and UC (n*=*242). A majority of the participants identified as female (302/461, 65.5%), white, non-Hispanic (342/461, 74.2%), having completed at least a 4-year college degree (307/461, 66.5%), married (314/461, 68.2%), being a full-time employee (309/461, 67.0%), being at least 40 years old (254/461, 55.1%), and as residing in a household with an annual income of at least US $75,000 (238/461, 51.6%). The difference in the proportion of missing data observed at T2 for the FFW group (ie, 0.12) as compared with the UC group (ie, 0.07) was not statistically significant (*P*=.08.)

Table 1 provides a comparison of demographic characteristics, BMI values, self-efficacy domain scores, and physical activity scores at T1 for participants by randomization group. There were no statistically significant differences in the proportions (for binary variables tested via logistic regression) or means (for continuous variables tested via linear regression) of demographic characteristics, the mean BMI value, the mean self-efficacy domain scores, or the mean physical activity scores at T1 by randomization group. The minimum value of BMI observed across the sample was 25.06 kg/m^2^. The median values of physical activity in hours per week (ie, 10.61 and 9.18) were similar to IPAQ-based values in some other relevant research [48-50]. No important harms or unintended effects were observed in either group. Cronbach alpha ranged from .86 (physical activity) to .97 (work-related physical activity self-efficacy). A majority (201/219, 91.7%) of the participants who were assigned to the FFW group were engaged with the FFW intervention.

**Table 1:** Descriptive statistics for demographic characteristics, self-efficacy domain scores, and physical activity scores at baseline for participants by randomization group (N=461).

Variable^a^	Usual care (n=242)	Fun for Wellness (n=219)
Female, n (%)	157 (64.9)	144 (66.0)
Black, n (%)	41 (16.9)	31 (14.2)
Hispanic, n (%)	17 (7.0)	13 (5.9)
Vocational or technical school, n (%)	17 (7.0)	15 (6.9)
Some college, n (%)	37 (15.3)	39 (18.0)
Undergraduate degree, n (%)	111 (46.0)	85 (38.7)
Graduate or professional degree, n (%)	54 (22.4)	56 (25.7)
Married, n (%)	165 (68.2)	149 (68.1)
Part-time employment, n (%)	28 (11.6)	19 (8.7)
Full-time employment, n (%)	158 (65.3)	151 (69.0)
Retired, n (%)	21 (8.7)	19 (8.8)
Age (years), mean (SD)	41.97 (11.03)	41.77 (10.78)
Income in thousand dollars, mean (SD)	76.38 (47.73)	77.77 (48.20)
BMI (kg/m^2^), mean (SD)	30.92 (5.83)	30.21 (5.31)
Work-related physical activity self-efficacy (alpha=.97), mean (SD)	1.19 (1.15)	1.17 (1.14)
Transport-related physical activity self-efficacy (alpha=.95), mean (SD)	1.20 (1.11)	1.28 (1.16)
Domestic-related physical activity self-efficacy (alpha=.95), mean (SD)	1.43 (1.22)	1.52 (1.25)
Leisure time–related physical activity self-efficacy (alpha=.97), mean (SD)	1.37 (1.17)	1.39 (1.25)
Self-efficacy to regulate physical activity (alpha=.90), mean (SD)	2.06 (0.74)	2.05 (0.70)
Physical activity in hours per week (alpha=.86), median (IQR)	10.61 (19.17)	9.18 (17.65)

^a^The reference group (eg, male) for each demographic variable (eg, gender) and subgroups comprising less than 5% of observations are not reported for spatial reasons. Missing data ranged from 0% to 3.5% across all the variables in this table.

### Path Model

**Figure 3:**
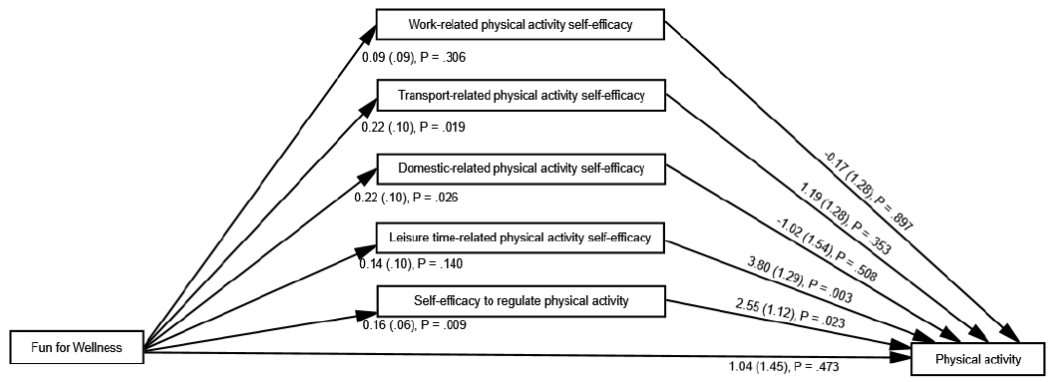
Key focal unstandardized parameter estimates from the path model for hypothesis 1 through hypothesis 3. The 241 nonfocal parameter estimates are not depicted to reduce clutter.

The percentage of variance accounted for ranged from 16.8% (work-related physical activity self-efficacy) to 25.3% (self-efficacy to regulate physical activity) across the five domains of self-efficacy at T2 and equaled 37.4% for physical activity at T3. The correlations among the residuals of the four self-efficacy–level constructs ranged from 0.74 (work-related physical activity self-efficacy with leisure-related physical activity self-efficacy) to 0.76 (transport-related physical activity self-efficacy with domestic-related physical activity self-efficacy). The correlations between the residuals of the four self-efficacy–level constructs with self-efficacy to regulate physical activity ranged from 0.04 (transport-related physical activity self-efficacy with self-efficacy to regulate physical activity) to 0.13 (work-related physical activity self-efficacy with self-efficacy to regulate physical activity). The unstandardized estimates of the covariates are available in Table 2, but these estimates are not discussed because of spatial limitations. Table 3 provides the unstandardized estimate of each focal parameter from the path model by hypothesis. Figure 3 provides key focal unstandardized parameter estimates for hypothesis 1 through hypothesis 3. Estimates for hypothesis 4 are not directly provided in Figure 3 because they are not parameter estimates per SE but rather a function of existing parameter estimates. However, they are listed at the bottom of Table 3. The paragraphs below briefly interpret these estimates with regard to the corresponding hypothesis tested.

**Table 2:** Unstandardized estimate of the covariates from the path model.

Predictor	Outcome					
	Work-related physical activity self-efficacy at time 2, beta (SE)	Transport-related physical activity self-efficacy at time 2, beta (SE)	Domestic-related physical activity self-efficacy at time 2, beta (SE)	Leisure-related physical activity self-efficacy at time 2, beta (SE)	Self-efficacy to regulate physical activity at time 2, beta (SE)	Physical activity at time 3, beta (SE)
Physical activity at time 1	.01 (0.00)^a^	.01 (0.00)^b^	.01 (0.00)^a^	.01 (0.00)^b^	.01 (0.00)^c^	.46 (0.07)^b^
Female	−.17 (0.10)	−.13 (0.09)	−.02 (0.10)	−.08 (0.10)	−.10 (0.07)	1.27 (1.24)
Black	−.23 (0.12)	−.15 (0.13)	−.04 (0.14)	−.11 (0.13)	.16 (0.09)	−.32 (1.73)
Hispanic	.00 (0.21)	−.15 (0.22)	−.30 (0.22)	−.35 (0.18)^c^	.12 (0.12)	−.65 (2.61)
Vocational or technical school	.03 (0.28)	.06 (0.28)	.16 (0.31)	.29 (0.30)	.22 (0.23)	5.86 (4.44)
Some college	−.27 (0.22)	−.15 (0.25)	.11 (0.27)	.02 (0.25)	−.03 (0.21)	2.05 (3.71)
Undergraduate degree	−.12 (0.22)	.12 (0.24)	.20 (0.26)	.30 (0.25)	.13 (0.21)	.39 (3.00)
Graduate or professional degree	−.52 (0.23)^c^	−.22 (0.25)	−.12 (0.27)	−.14 (0.26)	.20 (0.21)	3.36 (3.24)
Married	−.07 (0.12)	−.15 (0.13)	−.12 (0.13)	−.09 (0.13)	−.06 (0.08)	1.62 (1.69)
Part-time employment	.11 (0.25)	−.05 (0.26)	−.32 (0.27)	−.09 (0.26)	.27 (0.17)	2.41 (3.70)
Full-time employment	−.21 (0.22)	−.58 (0.22)^a^	−.91 (0.24)^b^	−.76 (0.23)^a^	.40 (0.15)^a^	−1.81 (3.47)
Retired	−.46 (0.25)	−.79 (0.26)^a^	−.86 (0.29)^a^	−.78 (0.27)^a^	.04 (0.22)	−2.28 (4.32)
Age in years	.02 (0.01)^b^	.02 (0.01)^b^	.03 (0.01)^b^	.03 (0.01)^b^	−.02 (0.00)^b^	.18 (0.09)^c^
Income in thousand dollars	.01 (0.00)^b^	.01 (0.00)^b^	.01 (0.00)^b^	.01 (0.00)^b^	.00 (0.00)	.02 (0.02)

^a^*P*<.01, 2-tailed.

^b^*P*<.001, 2-tailed.

^c^*P*<.05, 2-tailed.

**Table 3:** Unstandardized estimate of each focal parameter from the path model by hypothesis.

Specific path		Beta_1_ (SE)	95% CI	Cohen *d*	95% CI
**Hypothesis 1: FFW^a^ –> self-efficacy**					
	FFW **–>** work-related physical activity self-efficacy at time 2	.09 (0.09)	−0.09 to 0.27	0.10	−0.09 to 0.28
	FFW **–>** transport-related physical activity self-efficacy at time 2	.22 (0.10)^b^	0.04 to 0.41	0.23	0.04 to 0.41
	FFW **–>** domestic-related physical activity self-efficacy at time 2	.22 (0.10)^b^	0.03 to 0.41	0.22	0.03 to 0.40
	FFW **–>** leisure-related physical activity self-efficacy at time 2	.14 (0.10)	−0.05 to 0.33	0.14	−0.04 to 0.33
	FFW **–>** self-efficacy to regulate physical activity at time 2	.16 (0.06)^c^	0.04 to 0.29	0.25	0.07 to 0.43
**Hypothesis 2: Self-efficacy –> physical activity**					
	Work-related physical activity self-efficacy at time 2 **–>** physical activity at time 3	−.17 (1.28)	−2.68 to 2.35	—^d^	—
	Transport-related physical activity self-efficacy at time 2 **–>** physical activity at time 3	1.19 (1.28)	−1.32 to 3.6	—	—
	Domestic-related physical activity self-efficacy at time 2 **–>** physical activity at time 3	−1.02 (1.54)	−4.03 to 1.99	—	—
	Leisure-related physical activity self-efficacy at time 2 **–>** physical activity at time 3	3.80 (1.29)^c^	1.26 to 6.33	—	—
	Self-efficacy to regulate physical activity at time 2 **–>** physical activity at time 3	2.55 (1.12)^b^	0.35 to 4.76	—	—
**Hypothesis 3: FFW –> physical activity**					
	FFW **–>** physical activity at time 3	1.04 (1.45)	−1.80 to 3.88	0.07	−0.11 to 0.26
**Hypothesis 4: FFW –> self-efficacy –> physical activity**					
	FFW **–>** work-related physical activity self-efficacy at time 2 **–>** physical activity at time 3	−.02 (0.12)	−0.58 to 0.27	—	—
	FFW **–>** transport-related physical activity self-efficacy at time 2 **–>** physical activity at time 3	.26 (0.31)	−0.24 to 1.20	—	—
	FFW **–>** domestic-related physical activity self-efficacy at time 2 **–>** physical activity at time 3	−.22 (0.34)	−1.26 to 0.3	—	—
	FFW **–>** leisure-related physical activity self-efficacy at time 2 **–>** physical activity at time 3	.54 (0.41)	−0.06 to 1.76	—	—
	FFW **–>** self-efficacy to regulate physical activity self-efficacy at time 2 **–>** physical activity at time 3	.42 (0.25)	0.06 to 1.14^e^	—	—

^a^FFW: Fun for Wellness.

^b^*P*<.05, 2-tailed.

^c^*P*<.01, 2-tailed.

^d^Not applicable.

^e^Bias-corrected confidence interval did not include 0.

#### Hypothesis 1

The adjusted mean difference for the FFW group as compared with the UC group was statistically significant and approximately small in size for transport-related physical activity self-efficacy (beta=.22, *P*=.02; *d*=0.23), domestic-related physical activity self-efficacy (beta=.22, *P*=.03; *d*=0.22), and self-efficacy to regulate physical activity (beta=.16, *P*=.01; *d*=0.25) at T2. The adjusted mean difference for the FFW group as compared with the UC group was statistically nonsignificant for work-related physical activity self-efficacy (beta=.09, *P*=.31; *d*=0.10) and leisure-related physical activity self-efficacy (beta=.14, *P*=.14; *d*=0.14) at T2. Thus, only partial support was provided for hypothesis 1.

#### Hypothesis 2

The path coefficient to physical activity at T3 was statistically significant for leisure-related physical activity self-efficacy (beta=3.80, *P*=.003) and self-efficacy to regulate physical activity (beta=2.55, *P*=.02) at T2. The path coefficient to physical activity at T3 was statistically nonsignificant for work-related physical activity self-efficacy (beta=−.17, *P*=.90), transport-related physical activity self-efficacy (beta=1.19, *P*=.35), and domestic-related physical activity self-efficacy (beta=−1.02, *P*=.51) at T2. Thus, only partial support was provided for hypothesis 2.

#### Hypothesis 3

The adjusted mean difference on physical activity at T3 for the FFW group as compared with the UC group was statistically nonsignificant (beta=1.04, *P*=.47, *d*=0.07). Thus, no support was provided for hypothesis 3.

#### Hypothesis 4

The 95% CI for the product of path coefficients from FFW to physical activity at T3 through self-efficacy at T2 did not include 0.00 for self-efficacy to regulate physical activity (beta=.42, 95% CI 0.06 to 1.14). The 95% CI for the product of path coefficients from FFW to physical activity at T3 through self-efficacy at T2 included 0.00 for work-related physical activity self-efficacy (beta=−.02, 95% CI −0.58 to 0.27), transport-related physical activity self-efficacy (beta=.26, 95% CI −0.24 to 1.20), domestic-related physical activity self-efficacy (beta=−.22, 95% CI −1.26 to 0.36), and leisure-related physical activity self-efficacy (beta=.54, 95% CI −0.06 to 1.76). Thus, only partial support was provided for hypothesis 4.

## Discussion

### Principal Findings

The objective of this study was to evaluate the effectiveness of the FFW Web-based behavioral intervention to increase physical activity in adults with obesity in the United States in a relatively uncontrolled setting. In general, results from this study provide both some supportive and some unsupportive initial evidence with regard to the objective of this study. Specific findings, both supportive and unsupportive, will be discussed with respect to the four construct-level hypotheses tested within the FFW conceptual model for the promotion of physical activity (see Figure 1) and to the relevant results from the 2015 FFW efficacy trial.

Partial supportive evidence was observed in this study for three of the four hypotheses tested. Supportive evidence for hypothesis 1 includes positive direct effects from the FFW intervention to transport- and domestic-related physical activity self-efficacy and self-efficacy to regulate physical activity at T2. This set of findings provides some support for the conceptualization of the BET I CAN challenges as capability-enhancing opportunities and extends the literature on the ability of FFW to promote self-efficacy beliefs [3]—a potentially modifiable mediating variable targeted by the intervention. Supportive evidence for hypothesis 2 includes positive direct effects from both leisure-related physical activity self-efficacy and self-efficacy to regulate physical activity at T2 to physical activity at T3. This pair of findings provides some support for a central contention of the self-efficacy theory—behaviors are an omnibus outcome of self-efficacy beliefs [25]—and addresses a limitation of the 2015 FFW efficacy trial: not evaluating proposed relationships between self-efficacy and physical activity [2]. Supportive evidence for hypothesis 4 includes a positive indirect effect of the FFW intervention on physical activity at T3 through self-efficacy to regulate physical activity at T2. This finding addresses a limitation of the 2015 FFW efficacy trial: not evaluating the proposed positive indirect effect of the FFW intervention on physical activity through self-efficacy. Beyond the four hypotheses tested, this study has the potential to be important because it provides initial evidence for the effectiveness of the FFW intervention to increase physical activity (indirectly through self-efficacy to regulate physical activity) in an at-risk population [8,13]. Beyond the FFW intervention, findings from this study also contribute to a practical research need identified in the 2018 Physical Activity Guidelines Advisory Committee Scientific Report: to systematically test theory-based interventions in real-world settings [9].

At least partial unsupportive evidence was observed in this study for each of the four hypotheses tested. Unsupportive evidence for hypothesis 1 includes null direct effects from the FFW intervention to both work- and leisure-related physical activity self-efficacy at T2. Thus, it may be that the BET I CAN challenges in the FFW intervention would benefit from being further optimized for providing more meaningful exposure to relevant sources of efficacy-enhancing information with regard to these 2 domains of self-efficacy beliefs [51]. More specifically, future studies that estimate the individual effect of each BET I CAN component, and how BET I CAN components may operate synergistically with each other, may help identify active and inactive intervention components within FFW with regard to promoting self-efficacy and physical activity in adults with obesity. Unsupportive evidence for hypothesis 2 includes null direct effects from work-, transport-, and domestic-related physical activity self-efficacy at T2 to physical activity at T3. This set of null findings may be because of the relatively strong correlations among the four self-efficacy–level constructs (ie, difficult to identify unique relationships with physical activity). Unsupportive evidence for hypothesis 3 includes a null direct effect from the FFW intervention to physical activity at T3. This null finding is inconsistent with relevant results from the 2015 FFW efficacy trial [2] and may be because of differences in either model specification (ie, evaluating the direct effect of FFW on physical activity while controlling for self-efficacy beliefs in this study) or measurement of physical activity (ie, more thoroughly measuring physical activity in this study). Unsupportive evidence for hypothesis 4 includes null indirect effects from the FFW intervention to physical activity at T3 through each of the four self-efficacy–level constructs: work-, transport-, leisure-, and domestic-related physical activity self-efficacy at T2. This set of null findings may be attributable to the idea that, on average, an individual’s self-efficacy beliefs regarding their capability to engage in a recommended amount of physical activity for health may be less important than an individual’s self-efficacy beliefs in their capability to overcome possible barriers to their engagement in a recommended amount of weekly physical activity for health with regard to the promotion of an individual’s physical activity behavior [26,27].

### Conclusions

Results from this study provide some initial evidence for both the effectiveness and the ineffectiveness of the FFW Web-based behavioral intervention to increase physical activity in adults with obesity in the United States. Specifically, there is evidence that FFW may be ineffective in directly promoting physical activity in adults with obesity. Similarly, there is evidence that FFW may be ineffective in indirectly promoting physical activity through the four (ie, work-, transport-, domestic-, and leisure time–related) *self-efficacy–level constructs* (ie, the degree to which an individual perceives that they have the capability to engage in a recommended amount of weekly physical activity for health). However, there is evidence that FFW may be effective in indirectly promoting physical activity in adults with obesity by increasing an individual’s *self-efficacy to regulate their physical activity* (ie, the degree to which an individual perceives that they have the capability to *overcome possible barriers to* engagement in a recommended amount of weekly physical activity for health). For this reason, we believe that the FFW Web-based behavioral intervention may have the potential to eventually become useful, in some small but important way, given the magnitude of the problem, in responding to the global pandemic of insufficient physical activity in adults with obesity by increasing an individual’s self-efficacy to regulate their physical activity.

Realizing the potential for the FFW intervention to have practical implications at a local level will require future community-based studies that align with recent recommendations put forth by the Community Preventive Services Task Force [52]. More specifically, the Community Preventive Services Task Force suggests that physical activity interventions for adults with obesity should include activity monitors and promote physical activity within a more broadly focused weight management program where there is access to a health care provider. An implication from the results of this study is that a feasibility study is now underway to implement accelerometer-based assessment of physical activity within the FFW intervention in partnership with a local bariatric service center within a major health care organization in the Midwest of the United States [53]. Gaining necessary approvals for accessing medical records from participants in this ongoing feasibility study may provide important information on certain patient characteristics (eg, comorbidities) that may influence the effectiveness of the FFW intervention.

### Limitations

We are aware of at least four noteworthy limitations for this study that temper the relevant conclusions that can be made. First, we recognize that our hypotheses assume additivity of FFW effects for all covariates (ie, no a priori moderators for the proposed effects of FFW). We encourage future secondary analyses that explore the prospect of heterogeneous FFW effects for subgroups of individuals (eg, comorbidities) on physical activity. Second, we note that another limitation is that all the data collected, except for engagement with the FFW intervention, were collected via self-reporting. Field-based studies that collect physical activity data from objective instrumentation [54-58] in adults with obesity are encouraged [35,52] and are underway in the FFW context [53]. This underway study is employing both self-reported and accelerometer-measured physical activity in adults with obesity, which is consistent with recommendations in previous research [59] that found the physical activity of overweight or obese individuals to be ranked higher by self-reporting than by accelerometer as compared with normal-weight individuals. That said, it is important to note that the aforementioned published study did not provide evidence for randomized group assignment (eg, control vs experimental) as a moderator for the observed mismatch between self-reported and accelerometer-measured physical activity in overweight or obese individuals engaged in physical activity–promoting interventions. Thus, although the aforementioned study provides support for suspecting that a mismatch between self-reported and accelerometer-measured physical activity may have been observed in this study (if accelerometer-measured physical activity had been collected), it does not provide direct support for suspecting that the magnitude of the suspected mismatch may have varied as a function of randomized group assignment in this study (ie, UC group vs FFW group). The third limitation is that 360 of 820 cases (eg, 43.9%) needed to be excluded from the analyses because of either fraud (n=154) or outlying physical activity scores (n=206). Future efforts to better guard against fraud (eg, working more closely with the panel recruitment company) and possible overreporting of physical activity (eg, objective assessment of physical activity) is encouraged and may increase confidence in subsequent findings (eg, in reference to physical activity guidelines). A final limitation is that engagement data were not collected from UC participants who were given 1 month of 24-hour access to the FFW intervention (but were not provided with financial incentives to complete BET I CAN challenges) after data collection for this study was closed. Collecting these data would have provided some insight into the degree to which the very high level of engagement observed in the FFW group (ie, 201/219, 91.7%) may have been because of the inclusion of financial incentives to complete BET I CAN challenges.

## Appendix

Multimedia Appendix 1 CONSORT-EHEALTH checklist (V 1.6.1).

## Abbreviations

BARSE: barriers self-efficacy
BET I CAN: behaviors, emotions, thoughts, interactions, context, awareness, and next steps
EXSE: exercise self-efficacy
FFW: Fun for Wellness
IPAQ: International Physical Activity Questionnaire
IRB: institutional review board
ML: maximum-likelihood
T1: baseline
T2: 30 days after baseline
T3: 60 days after baseline
UC: usual care
VPS: virtual private server
WHO: World Health Organization
